# Screen-Printed Electrode-Based Sensors for Food Spoilage Control: Bacteria and Biogenic Amines Detection †

**DOI:** 10.3390/bios10100139

**Published:** 2020-09-30

**Authors:** Ricarda Torre, Estefanía Costa-Rama, Henri P. A. Nouws, Cristina Delerue-Matos

**Affiliations:** 1 REQUIMTE/LAQV, Instituto Superior de Engenharia do Porto, Instituto Politécnico do Porto, Dr. António Bernardino de Almeida 431, 4200-072 Porto, Portugal; rdvdt@isep.ipp.pt (R.T.); han@isep.ipp.pt (H.P.A.N.); 2 Departamento de Química Física y Analítica, Universidad de Oviedo, Av. Julián Clavería 8, 33006 Oviedo, Spain

**Keywords:** screen-printed electrode, electroanalysis, electrochemical sensor, biosensor, immunosensor, food analysis, bacteria, biogenic amines, histamine

## Abstract

Food spoilage is caused by the development of microorganisms, biogenic amines, and other harmful substances, which, when consumed, can lead to different health problems. Foodborne diseases can be avoided by assessing the safety and freshness of food along the production and supply chains. The routine methods for food analysis usually involve long analysis times and complex instrumentation and are performed in centralized laboratories. In this context, sensors based on screen-printed electrodes (SPEs) have gained increasing importance because of their advantageous characteristics, such as ease of use and portability, which allow fast analysis in point-of-need scenarios. This review provides a comprehensive overview of SPE-based sensors for the evaluation of food safety and freshness, focusing on the determination of bacteria and biogenic amines. After discussing the characteristics of SPEs as transducers, the main bacteria, and biogenic amines responsible for important and common foodborne diseases are described. Then, SPE-based sensors for the analysis of these bacteria and biogenic amines in food samples are discussed, comparing several parameters, such as limit of detection, analysis time, and sample type.

## 1. Introduction

The impact of food contamination by microorganisms and other poisonous substances is considered a major public health and safety concern. According to the World Health Organization (WHO), each year 600 million people (almost 1 in 10) fall ill because of contaminated food [1]. Pathogens have the ability to adapt to various environments, causing contaminations in different stages of the food production and supply chains. Thus, they can appear in raw food but also at any point of the food production process and even after the consumer acquires the food if the necessary precaution to transport and store is not taken. Many microorganisms are affected by heat and can be destroyed or inactivated after cooking [2,3]. However, some of them, and substances such as histamine (the main biogenic amine), are not affected by cooking, freezing, or canning processes [3,4]. Taking this into account, the importance of the control of contamination along the whole food chain is clear. Analytical methods and devices for real-time control of food safety and quality provide immediate information that allows corrective actions to be taken before the food products are made available for consumption.

Among the microorganisms that cause foodborne illnesses, bacteria are the most important because of their high occurrence [1,5]. These bacteria can be detected by appropriate techniques and methods such as cell culture and colony counting, polymerase chain reaction (PCR) and immunological assays [3,6]. Biogenic amines are nitrogenous species usually present in different foods that, at normal levels, do not entail health risks. However, their levels increase when food, especially fish, is stored for a long time and/or at an inadequate temperature (>4 °C) [7,8,9]. Therefore, the quantification of biogenic amines, especially histamine, is included in the routine analysis of many food industries. The analysis of biogenic amines is often performed through chromatographic methods (mainly liquid) coupled to different detectors [10]. Enzymatic kits are also employed since they are simpler and require cheaper instrumentation [11].

**Figure 1:**
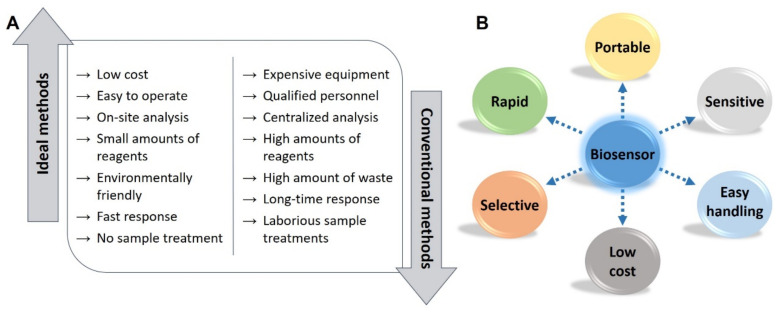
Schematic representation of (**A**) the advantageous features of ideal analytical methods vs. disadvantageous features of conventional ones and (**B**) the advantages of screen-printed based biosensors. The last one adapted from [12] with permission from Elsevier.

Although the above-mentioned methods (i.e., cell culture and colony counting, PCR, chromatography) are very useful, robust and provide accurate results, they are time consuming, involve complex processing steps and require highly trained analysts and expensive/complex instrumentation (Figure 1A). Therefore, the analyses have to be performed in centralized laboratories and the results are not available in real-time. Taking into account the time the different steps of the analytical process take (sampling, sample preparation, analysis, results interpretation, and communication) and the short shelf life of food products, the development of analytical methods that allow rapid screening of pathogens and spoilage indicators is critical to ensure food safety.

In this context, electrochemical (bio)sensors based on screen-printed electrodes (SPEs) have gained increasing interest as analytical tools for food analysis since SPEs provide great advantages that make these kind of sensors have the important characteristics of ideal biosensors (Figure 1B) [12]: ease of use, low-cost, and portability [13,14]. So, the screen-printed technology has significantly contributed to the transition from the traditional unwieldy electrochemical cells to miniaturized and portable electrodes that meet the needs for on-site analysis [12,15]. Although a screen-printed electrode (SPE) is not as robust as a conventional electrode, such as glassy carbon or gold disk, and the surface of its working electrode is not as perfect as the one of a mirror-like polished solid electrode, the advantages of SPEs regarding cost and size led to their increasing use in the last years as transducers in (bio)sensing. The use of SPE-based sensors in the control of food spoilage as complementary analytical tools to the conventional methods allows a rapid screening at any point of the food production chain, preventing the occurrence of foodborne illnesses and the reduction of food waste.

The purpose of this article is to review SPE-based biosensors for the analysis of bacteria and biogenic amines related with food spoilage, focusing on the analyte, and discussing the different approaches and trends in the development of these sensors. The main characteristics of SPEs as transducers and the main challenges on improved SPE-based biosensors are also highlighted.

## 2. Screen-Printed Electrodes as Transducers

### 2.1. Production and Design of Screen-Printed Electrodes

**Figure 2:**
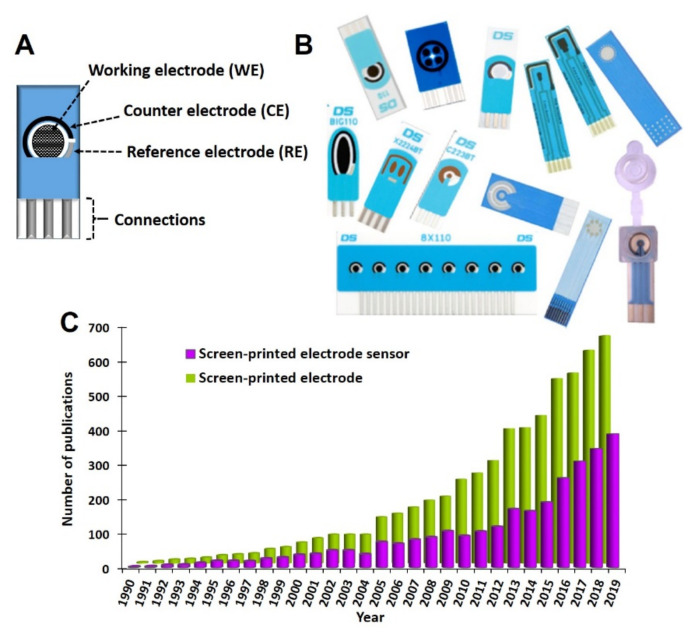
(**A**) Scheme of the most common configuration of a screen-printed electrode. (**B**) Examples of commercial screen-printed electrodes with different configurations and designs. Reproduced from [19] with permission from Wiley. (**C**) Number of publications per year when searching “screen-printed electrode” and “screen-printed electrode sensor” in Scopus database for the last 30 years (1990–2019).

The screen-printing technology was adapted from the microelectronics industry and is used, among others, to produce screen-printed electrodes (SPEs) (Figure 2A,B). These electrodes offer the main characteristics required to obtain electrochemical sensing platforms for on-site analysis. Although this technology exists in its present form since the 20th century [16], it began to be used for the fabrication of electrochemical cells in the 1990’s. Since then, the use of SPEs as transducers for many different electrochemical sensors has steadily increased (Figure 2C). Nowadays, the screen-printing technology is a common and well-established technique for the conception of electroanalytical devices with assorted applications: from point-of-care (POC) devices for biomedical applications [17,18,19] to portable sensors for food analysis [13,14] and detection of environmental contaminants [12,20,21]. SPEs usually contain an electrochemical cell composed of three electrodes (working-WE-, reference-RE-, and counter-CE-electrodes) printed on a solid substrate (Figure 2A). Different inks (the most common are carbon and metallic inks) to print the electrodes [22] and different substrates (often ceramic or plastic) can be used. The SPE’s fabrication process is fast and allows large scale and highly reproducible production of small-sized, cheap, and disposable electrodes. Therefore, it is not necessary to clean and/or polish them, avoiding tedious pretreatment steps, saving a lot of time. In contrast, the robustness of the printed electrodes and their electrochemical features are not as good as those of conventional electrodes (e.g., glassy carbon, gold disk, etc.). However, SPEs show adequate electroanalytical features for sensing applications and this, together with their low-cost and ease of use (which avoids the need of highly skilled analysts) make SPEs clearly advantageous as transducers for applications in which on-site one-point measurements are required. Moreover, the miniaturized design of SPEs not only allows to transport them to perform on-site measurements for real-time analysis, but also avoids the use of high amounts of reagents and samples. All these characteristics are in accordance with the principles of Green Analytical Chemistry [23,24].

Briefly, the fabrication of SPEs consists of the following steps: (i) design of the screen or mesh that will define the geometry and size of the SPE; (ii) selection and preparation of the conductive inks and selection of the substrate material; (iii) layer-by-layer deposition of the chosen inks on the solid substrate and (iv) drying and curing [17,20]. By covering the electrical circuits with an insulating material it is possible to perform the analytical measurement by depositing a single drop of the reagent/sample solution onto the SPE, by immersing it into a solution or by including it in a flow system. Regarding the inks for the WE, as mentioned before, the most popular ones are based on carbon (graphite, graphene, fullerene, carbon nanomaterials, etc.) because of their suitable features for electroanalysis (i.e., good conductivity, chemical inertness, ease of modification, low background currents, and a wide potential range) and their low costs [17,25]. Besides carbon inks, conductive metallic inks have increasingly been used; among them gold ink is the most common due to its high affinity with thiol moieties that allows easy surface modification with proteins by the formation of self-assembled monolayers (SAMs). SPEs with a WE made of other metallic inks such as silver, platinum or palladium are also available on the market [26] but their use is scarce and limited to specific applications. The use of SPEs with an optically transparent WE, made of indium tin oxide (ITO), PEDOT or even gold (obtained by sputtering process) or carbon (made of carbon nanotubes), is increasing because of the growing interest in spectroelectrochemistry [26,27,28,29,30,31]. The RE is often made of silver or silver/silver chloride ink. This is considered a pseudo-reference or quasi-reference electrode since its potential is not as stable as that of an ideal reference electrode (e.g., conventional silver/silver chloride RE, which is the most common). Therefore, the applied potential is not as exact and reproducible as when an Ag/AgCl electrode is used. This can be problematic for electrochemical studies in which the control of the potential is essential; however, for sensing applications, this is rarely a problem. The CE is usually made of the same ink as the WE. Because the composition of the inks defines the electrochemical characteristics of the electrode, SPEs are highly versatile. However, the versatility of SPEs is not only due to the use of different inks, but also because of the ease of modification of the WE. The purpose of these modifications is to enhance the electroanalytical characteristics of the SPEs (such as sensitivity, precision, operational stability) and to improve the immobilization of the recognition element (which are often biological elements (e.g., proteins, DNA, etc.), but can also be synthetic (e.g., molecularly imprinted polymers (MIPs), see Section below)) [17]. For example, great enhancements of the analytical features have been achieved by using carbon nanomaterials (nanotubes, nanofibers, graphene, among others) and metallic nanoparticles (primarily gold nanoparticles, since they are cheaper than a WE made of gold ink) [18,32]. Besides these nanomaterials many other materials can be used: redox mediators, polymers, complexing agents, metallic oxides, etc. The simplest procedure to modify SPEs is by deposition of the modifying agent onto the WE; this procedure is facilitated because of the planar nature of the SPE, so it can be performed through an automatic dispenser in a mass-producible way. However, the WE of an SPE can also be modified by adding the modifier to the ink before printing, by chemical adsorption or by electrochemical deposition (a good example is the in-situ generation of metallic nanoparticles) [32,33,34].

Another source of the SPE’s versatility is the possibility of printing the electrochemical cell on a wide variety of substrates. The choice of the substrate will determine the stability, robustness, disposability, and applicability of the SPE. As mentioned before, the most common are rigid substrates such as ceramics. However, although printing the electrodes on non-planar and non-rigid surfaces is not so easy as on rigid ones, there are several works describing SPEs that were fabricated using paper sheets, cloths, stretch and foldable films, and even epidermis [18,20,25,35,36,37,38]. To choose the correct substrate, it is important to keep the final application in mind: for example, ceramics are easy to print on and are highly robust but are more expensive than paper. Paper is light and easy to transport but its flexibility and moisture tolerance is limited. Polymeric substrates, especially flexible ones, are interesting for wearable sensors; in these cases, the limitation is related to the bending endurance of the printed electrodes.

So, SPEs offer numerous advantages, but the most important one is their high adaptability. This adaptability is not confined to the materials to fabricate them (inks and substrates); it also covers their design. As said before, the most common option is printing one electrochemical cell (with three electrodes) on the substrate, but many others configurations are possible: SPEs with more than one WE sharing the same RE and CE, platforms with several complete electrochemical cells, 96-well SPE plate or even SPEs with an integrated micro-well/reactor [20,26,39,40] (Figure 2B). Thus, their high versatility together with their ease of use and portability make SPEs one of the main transducers for the development of electroanalytical devices.

### 2.2. (Bio)Sensors Based on SPEs

**Figure 3:**
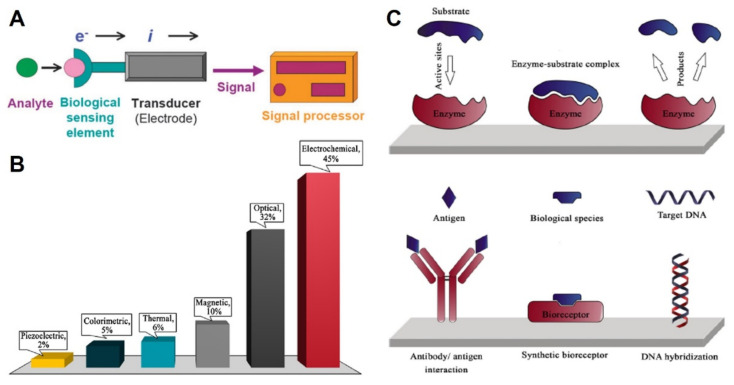
(**A**) A schematic representation of a biosensor with electrochemical transduction. Reproduced from [43] with permission from The Royal Society of Chemistry. (**B**) Distribution of different types of techniques for signal transduction using biosensors (data retrieved from the Scopus database from 2017 to August 2019). Reproduced from [25] with permission from Springer 2019. (**C**) Schematic illustration of enzymatic reaction on catalytic-based biosensors (top) and three different types of affinity-based biosensors (bottom). Reproduced from [25] with permission from Springer 2019.

As mentioned before, there are a great amount and variety of (bio)sensors based on SPEs with applications in very different fields. A biosensor is a type of chemical sensor; it can be defined as an analytical device able to provide (bio)chemical information, usually the concentration of a substance in a complex matrix, which consists of two main parts: a biological recognition element that selectively identifies the analyte of interest, and a transducer that transforms that recognition event into an measurable signal (Figure 3A) [41,42]. A biosensor should therefore contain biological elements that can be, mainly, (i) enzymes (catalytic biosensors) and (ii) proteins (antibody or antigen), or DNA or RNA strands (affinity biosensors) (Figure 3C). However, because of the advantages of artificial biomimetic receptors, such as MIPs and aptamers, regarding physical and chemical stability, it is increasingly accepted to include them in the “biosensor” category [25].

The most common SPE-based biosensors for food analysis are enzymatic- and immunosensors. Enzymatic biosensors are based on the highly selective interaction of the target analyte with an enzyme through its active sites, forming a complex that transforms the analyte into a (or more than one) product(s) [25,44]. The determination of the analyte is usually carried out by measuring the amount of generated product. Nevertheless, since co-factors or other co-reagents are sometimes needed, their consumption can be also used to monitor the analyte–enzyme interaction. Immunosensors are based on antibody–antigen interactions and take advantage of the high specificity of an antibody towards the corresponding antigen. In these sensors, the target analyte can either be the antibody or the antigen. Briefly, there are two main ways of following the immunoreaction: (i) using a label attached to a one of the immunoreagents, e.g., an enzyme or a nanoparticle that produces a detectable signal, and (ii) through label-free detection; in this case, the formation of the immunocomplex (antibody–antigen) produces a detectable physical/chemical change [14,19]. Immunosensors are highly specific and can be applied to a wide variety of analytes provided that an antibody that interacts with the analyte is available. Moreover, different strategies (e.g., the use of different labels or nanomaterials) can be used to improve their sensitivity. However, compared to enzymatic sensors, immunosensors are usually more labour intensive and less robust since several steps with long incubation times are required.

Independent of the type of recognition element, they have to be immobilized on the surface of the WE. The versatility of SPEs allows to choose between many different immobilization procedures: from the simplest one, the direct adsorption of the receptor by incubating it on the WE surface, to others that require more steps such as crosslinking, SAM formation, covalent binding, entrapment, or affinity binding (e.g., using the avidin-biotin system). By taking advantage of the transducer, immobilization of the recognition element through electrodeposition is also possible (a good example is the case of electrogenerated MIPs [45]). These immobilization methods are extensively described in several previous reviews [32,44,46,47,48].

When using biosensors, mainly electrochemical techniques are used for signal transduction, but colorimetric (without instrumentation), optical, magnetic, piezoelectric, and thermal techniques can also be employed (Figure 3B) [25,49]. In electrochemical biosensors, the analytical signal can be provided by different techniques: amperometry and voltammetry (based on current measurement), electrochemical impedance spectroscopy (EIS), potentiometry or conductometry [43,50,51]. The amperometric and voltammetric sensors are the most widely used because of their simplicity and applicability. Nevertheless, EIS sensors are gaining interest since there is no need for labels (especially used in immunosensing), but their sensitivities are often lower than the amperometric and voltammetric sensors [43].

## 3. Parameters Related to Food Spoilage

The production of safe and high-quality foodstuffs requires the control of several parameters at different points of the food production and supply chain: the quality of raw ingredients, the hygienic conditions of food production, the suitability of storage conditions, and the nutritional properties of the finished products [2]. Inadequate conditions at any stage of this chain often lead to food spoilage, involving chemical and physical changes (e.g., oxidation, colour changes, nasty smells, physical damages, etc.). Although food spoilage can be originated by various causes, the growth of microorganisms is the most common [1,5]. Many factors can contribute or accelerate food deterioration, such as exposure to inadequate levels of oxygen, moisture, light, or temperature. The microorganisms responsible for food spoilage include several bacteria, viruses, moulds, and yeasts. Among these, the bacteria *Salmonella*, *Escherichia coli*, *Campylobacter*, and *Listeria* are the most common foodborne pathogens [1,34]. Besides microorganisms, mycotoxins, which are toxic metabolites produced by fungi, are also important causes of foodborne illnesses [1]. Another common parameter to evaluate food safety and freshness is the level of biogenic amines, which are produced by the microbial decarboxylation of amino acids present in some foodstuffs such as fish, meat, and fermented foods [10,52,53].

### 3.1. Bacteria

Bacteria are the most common cause of foodborne illnesses. It is often difficult to notice their presence at low but harmful levels since visual or olfactory changes are not always easy to observe. Consequently, the consumer can ingest food contaminated with bacteria without realising it, causing illnesses with important implications. Although there are a great variety of bacteria responsible for foodborne illnesses, *Campylobacter*, *Escherichia coli*, *Salmonella*, and *Listeria* are the most common causes [1,5,42,54,55]. Therefore, these bacteria will be focussed on in this review.

*Campylobacter* are Gram-negative bacteria that live as commensals organisms in the gastrointestinal tract of humans and many domestic animals [56,57]. Campylobacteriosis is the most commonly reported gastrointestinal infection in humans in the EU [54,55]. It normally produces symptoms such as diarrhoea and vomiting that can last from 2 to 10 days. Its main food sources are undercooked meat (specially poultry), unpasteurized milk and vegetables [3,58]. Within the genus *Campylobacter*, the species *C. jejuni* is responsible for more than 80% of *Campylobacter* infections [3].

*Escherichia coli* (*E. coli*) are Gram-negative bacteria belonging to the *Enterobacteriaceae* family that inhabit the gastrointestinal tract of humans and warm-blooded animals. As commensal microorganism, *E. coli* live in mutually beneficial association with its host without causing diseases [59,60]. However, there are several *E. coli* strains with virulent attributes associated mainly with three clinical syndromes: diarrhoea, urinary tract infections or meningitis. [3,60]. Its ease of handling and the availability of its complete genome sequence makes *E. coli* an important microorganism in biotechnological, medical and industrial applications [59]. Among the intestinal pathogenic *E. coli* there are six well-describes variants known as pathovars or pathotypes: Enteropathogenic *E. coli* (EPEC), Enterotoxigenic *E. coli* (ETEC), Enteroaggregative *E. coli* (EAEC), Enteroinvasive *E. coli* (EIEC) and Diffusely adherent *E. coli* (DAEC) and Shiga toxin-producing *E. coli* (STEC, which includes the Enterohemorrhagic *E. coli*-EHEC-) [3,60]. Foodborne illness outbreaks related with *E. coli* can be associated with many types of food: from meats and unpasteurized milk or fruit juice to vegetables such as lettuce and spinach [3]. Symptoms of *E. coli* infections can be minor for some people; however, sometimes the infection may become a life-threatening illness causing serious problems such as kidney failure. STEC is the third most common cause of foodborne zoonotic illness [54]. *E. coli* O157:H7 currently accounts for most of the EHEC infections worldwide [3].

*Salmonella* are Gram-negative bacteria that belong to the *Enterobacteriaceae* family and is classified into two species: *S. bongori* and *S. enterica*. The latter are associated with the main public health concern [3,61]. Based on the Kaufmann-White scheme, *Salmonella* spp. are subdivided into serotypes and consequently, they are usually referred to by their serotype names [61]. Within *S. enterica*, which includes more than 2500 serotypes, *S.* Typhi and *S*. Paratyphi are responsible for typhoid illness (typhoid and paratyphoid fever, respectively) characterized by fever, headache, abdominal pain, and diarrhoea which can be fatal if suitable treatment is not provided [3,42,61,62]. Besides typhoidal illness, the other *Salmonella* serotypes can cause gastrointestinal illness (salmonellosis) that is less serious and its symptoms normally last for a few days [3]. In 2018, nearly 30% of the total foodborne illness outbreaks reported in the EU (5146 outbreaks affecting 48,365 people) were caused by *Salmonella* [54]. These outbreaks were mainly linked to eggs [54], however salmonellosis can also occur by the ingestion of other animal-derived contaminated foods such as milk, meat, or poultry, or even of contaminated fruits or raw vegetables [3,63].

*Listeria* are Gram-positive bacteria that comprise seventeen species, including *Listeria monocytogenes*, which is responsible for Listeriosis that, although presenting a low incidence, leads to high hospitalizations and mortality rates [50,55,64]. *L. monocytogenes* is highly persistent: it is salt-tolerant and can survive, and even grow, at temperatures below 1°C unlike many other pathogens [3]*. Listeria* can grow in several kinds of foods: raw milk, smoked fish, meats, and raw vegetables [3,50].

The main methods for the detection of these pathogens in foods are based on culturing and colony counting, which are characterized by laborious and time-consuming procedures, consumption of high amounts of reagents and the need for highly-trained personnel [3,5]. Alternative methods are those based on polymerase chain reaction (PCR) or real-time (quantitative) PCR that considerably reduce the analysis time (24 h or 3–6 h, respectively) but also involve laborious procedures [3,5,6,42]. Other detection methods are those based on immunoassays. Among them, enzyme-linked immunosorbent assays (ELISA) are the most common since their commercialization as kits facilitate their use and large-scale application [5,50,51].

### 3.2. Biogenic Amines

Biogenic amines (BAs) are nitrogenous low-molecular weight compounds that are mainly produced by the microbial decarboxylation of amino acids. There are eight BAs commonly present in animals, plants, and foods and can be classified in three groups based on their structure: (i) aliphatic (putrescine, cadaverine, spermine, and spermidine); (ii) aromatic (tyramine and phenylethylamine); and (iii) heterocyclic (histamine and tryptamine) (Table 1) [7,8,9,52]. BAs are important in several physiological processes, such as neuromodulating functions, and each one of them has key roles in organisms [7]. For example, histamine acts as a neurotransmitter, is related with intestinal physiological functions, and is involved in allergic reactions; tyramine has antioxidant effects, and putrescine is an important constituent of all mammalian cells [52,65].

In suitable levels BAs have beneficial effects, but the consumption of an excessive amount of BAs can be toxic to humans. The most common example is histamine fish poisoning (also known as scombroid poisoning) which is generally caused by the consumption of fish with high levels of histamine. The symptoms are headache, gastrointestinal, and skin problems, and their severity depends on the dosage [4,7,9]. The rapid increase of the concentration of histamine in fish is induced by unsuitable storage conditions (mainly temperatures >4 °C and long storage times) [4,7]. Fish with high levels of histidine (such as sardine or tuna) are more prone to develop histamine than histidine-poor fish. Moreover, histamine shows a high temperature stability, so it is not affected by cooking or freezing nor by sterilization or canning processes [4,7]. Thus, the concentration of histamine is a common parameter that is used in the fish industry as a quality and freshness indicator.

Although histamine is the main BA of concern due to its toxicity, the other BAs can also induce harmful effects on human health; for example, tyramine, phenylethylamine, and tryptamine cause hypertension, and putrescine and cadaverine can cause hypotension and bradycardia, and potentiate the toxicity of other amines, especially of histamine [7,8,66,67].

Besides fish and sea-food, BAs are found in several daily-life foodstuffs (wine, beer, cheese, other fermented foods and meat) [9,10,66]. Because of their microbiological origin, the concentration of BAs has been used for the assessment of the freshness of certain foods. With this aim, the biogenic amine index (BAI) has been proposed; this parameter can include different BAs depending on the type of food to be evaluated [10]. The most widely used BAI includes histamine, cadaverine, putrescine, and tyramine. A BAI lower than 5–10 mg/kg indicates a good quality and fresh food [8,52,66].

Hence, the concentration of BAs in food is an important parameter to control. The main methods for their quantification are based on chromatographic techniques combined with different extraction techniques such as solid phase extraction, ultrasound-assisted extraction or dispersive liquid–liquid microextraction [10,52,66,67]. Regarding chromatographic techniques, the most common is liquid chromatography (LC) combined with ultraviolet or fluorescence detectors (in which the BAs need to be derivatized since they exhibit neither UV absorption nor fluorescence emission), or tandem mass spectrometry [8,10,52,65,66,67].

## 4. SPE-Based (Bio)Sensors for the Determination of Food Spoilage Parameters

When a bibliographic search for articles about SPE-based sensors for determining parameters related to food spoilage is performed, a considerable number of works is found. However, this number is lower than for clinical or biomedical applications. Therefore, the development of these kind of sensors in this area will surely continue to be explored in the next years. Among the SPE-based sensors, there are several enzymatic- and immunosensors, but aptasensors were also described. The electroactivity of some of the analytes is also explored in some studies, avoiding the use of a recognition element. In the following section, different SPE-based sensors for the determination of important food spoilage parameters are discussed. The classification of these sensors is based on the analytes and mainly focuses on those with applications in food analysis.

### 4.1. SPE-Based (Bio)Sensors for Bacteria Detection

**Figure 4:**
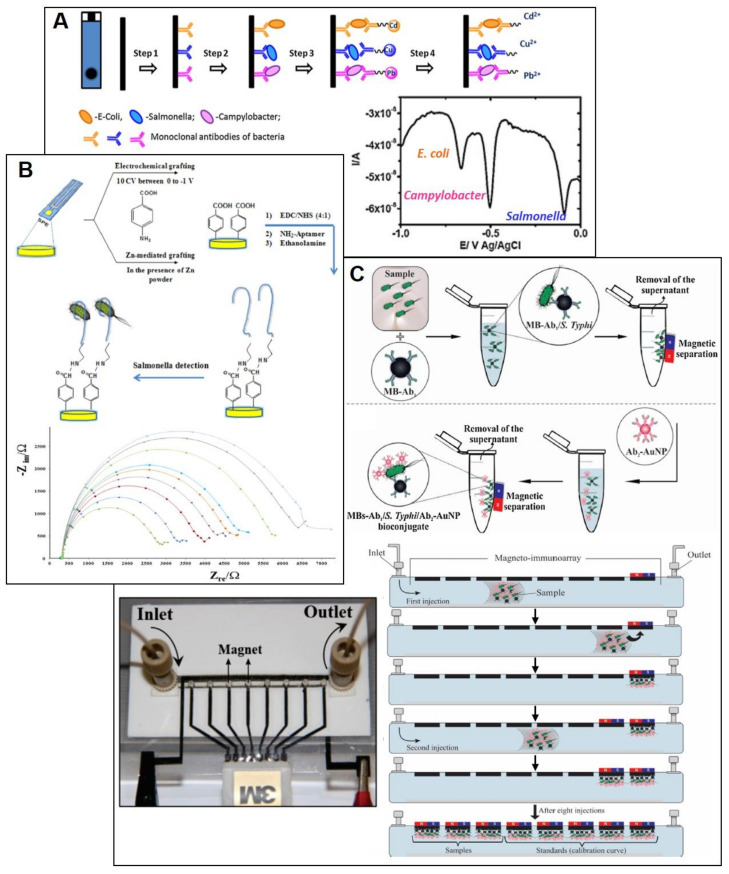
(**A**) Schematic representation of the multiplexed immunosensor developed by Viswanathan et al. for *E. coli*, *Salmonella*, and *Campylobacter* detection and the analytical signals obtained for the three bacteria by square-wave anodic stripping voltammetry (SWASV). Reproduced from [78] with permission from Elsevier. (**B**) Schematic representation of the aptasensor developed by Bagheryan et al. for *Salmonella* detection and the electrochemical impedance spectroscopy (EIS) signals obtained for different *Salmonella* concentrations included in the calibration curve. Reproduced from [73] with permission from Elsevier. (**C**) Schematic representation of the immunoassay based on magnetic beads developed by T.R. de Oliveira et al. using the microfluidic multiplex system shown in the picture. Reproduced from [75] with permission from Elsevier.

As mentioned before, among the bacteria responsible for foodborne diseases the main contributors, because of their incidence and the illnesses they cause, are *Campylobacter*, *Salmonella*, *E. coli*, and *Listeria*. Because of this, numerous biosensors have been developed for the determination of these microorganisms in food, as a whole or through target indicators of their presence (for example, specific cell membrane proteins or toxins). The wide incidence of salmonellosis has led to the development of many biosensors for the determination of *Salmonella* in foods such as milk or chicken meat (Table 2): most of them are immunosensors, for the serotype *Salmonella* Typhimurium, and based on SPEs with a carbon WE, both unmodified [68,69] or modified with nanomaterials [70,71,72], polymers [70], or an ionic liquid [71]. Although immunosensors are the main type of sensors, an aptasensor for *Salmonella* detection in apple juice is also reported [73] (Figure 4B). This is a label-free impedimetric sensor that used an SPCE modified with diazonium salt through chemical grafting on which the aminated-aptamer is immobilized. With this approach a concentration range between 10^1^ and 10^8^ CFU/mL and a limit of detection (LOD) of 6 CFU/mL was achieved. Several sensors use magnetic beads on which either the capture or the detection antibody is immobilized. A good example of this is the one developed for Ngoensawat et al. [74] in which the monoclonal capture antibody is immobilized on carboxylic acid-modified Fe_2_O_3_ magnetic particles on which multiwalled carbon nanotubes (MWCNT) modified with Methylene blue (the detection label) are immobilized. Once the immunomagnetic separation of *Salmonella* from the sample is performed, the detection is carried out through a sandwich type assay on an avidin-modified SPCE on which a biotin-labelled polyclonal antibody is immobilized. Using DPV as detection technique, a good LOD in milk samples is obtained: 17.3 CFU/mL. As in this case, using labels for monitoring the immunoaffinity event is the most common: the enzyme horseradish peroxidase is widely used. However, the use of nanomaterials such as gold nanoparticles (AuNP) [69,75,76] and CdS nanocrystals [68] is also frequent. A remarkable label is the one based on a polymeric dendron modified with CdTe Quantum Dots (QD) which was recently developed by Murasova et al. [77]. Using a specific anti-*Salmonella* antibody modified with this label, a sandwich immunoassay is performed using an antibody attached to magnetic beads. The detection is carried out through square-wave anodic stripping voltammetry (SWASV) on an SPCE modified with an on-site generated bismuth film obtaining a LOD of 4 CFU/mL. Viswanathan et al. explored the fact that metals show different redox potentials by using different metallic nanoparticles (CuS, CdS and PbS) to construct a multiplexed immunosensor for the simultaneous determination of *Salmonella*, *E. coli*, and *Campylobacter* [78]. The sensor consisted of a MWCNT-polyallylamine-modified SPCE on which specific antibodies for each one of the bacteria are immobilized. The sandwich is formed with detection antibodies specifically labelled with each one of the three different nanocrystals (Figure 4A). Using SWASV as technique detection, calibration curves in the range 10^3^–5 × 10^5^ cell/mL, and LODs of 400 cell/mL for *Salmonella* and *Campylobacter* and 800 cell/mL for *E. coli* are obtained. Another approach to develop sensors able to perform simultaneous measurements is the use of SPEs with more than one WE or more than one electrochemical cell. Examples of these sensors for *Salmonella* detection are also reported: from SPEs with two WEs [79], to a microfluidic system with eight WEs [75] (Figure 4C) or a 96-well-SPE plate [80].

For *Listeria* detection in food samples scarce works were found. A noteworthy example is the one developed by Tolba et al. [87] that used the cell wall binding domain (CBD) of bacteriophage-encoded peptidoglycan hydrolases (endolysin) as biorecognition element. CBD500 was immobilized by covalent binding on an SPE with a gold WE. After the reaction with the *Listeria* present in the sample, the analytical signal was obtained by EIS using [Fe(CN)_6_]^3−/4−^ as redox probe.

**Figure 5:**
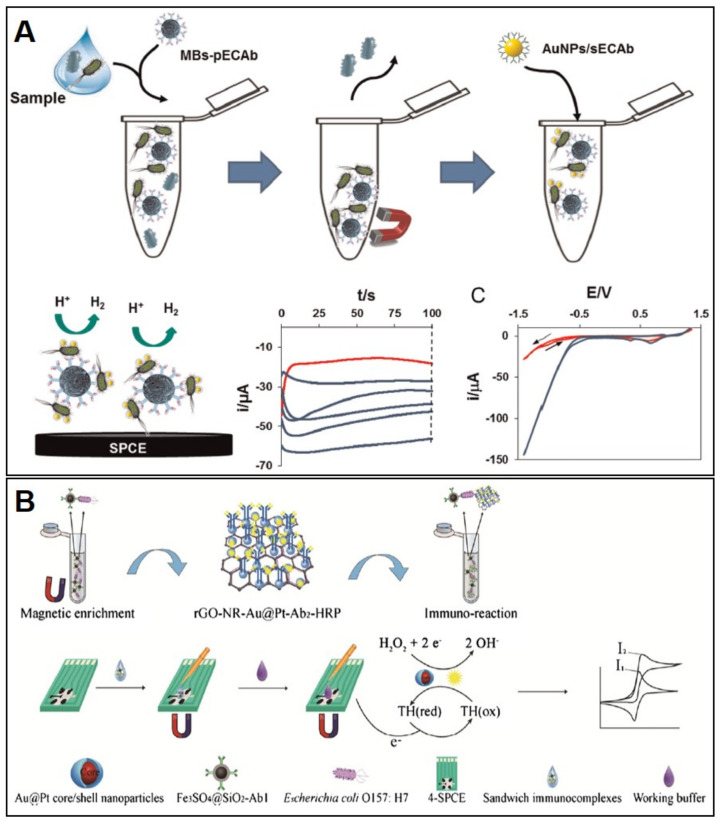
(**A**) Schematic representation of the magneto immunoassay developed by Hassan et al. for *E.coli* O157:H7 detection based on the Hydrogen Evolution Reaction electrocatalyzed by AuNP; chronoamperograms for different bacteria concentration; cyclic voltammograms in absence (red line) and presence of bacteria (blue line). Reproduced from [89] with permission from Elsevier. (**B**) Schematic representation of the magneto immunoassay, using rGO-NR-Au@Pt nanocomposite and HRP as label, developed by Wenchao Dou et al. *E. coli* O157:H7 detection. Reproduced from [92] with permission from Springer 2018.

As in the case of *Salmonella*, the SPE-based sensors for *E. coli* detection in foods are mostly based on immunoassays (Table 3). A simple biosensor was developed by Yueh-Hui Lui et al. that consisted of a sandwich immunoassay. The capture antibody was immobilized on an SPCE modified with AuNP and ferrocene dicarboxylic acid (FeDC) [88]. The detection antibody was labelled with HRP and H_2_O_2_ was used as substrate. The combination of AuNP and FeDC resulted in a significant improvement of the current intensity (studied by CV) when compared with an SPCE that was modified only with AuNP or FeDC. The SPCE contained two electrodes, both made of carbon ink: one acting as working and the other acting as both reference and counter electrodes. Using chronoamperometry (at 300 mV vs. carbon counter/reference electrode) as detection technique, the obtained immunosensor showed a quite good LOD of 600 CFU/mL. A lower LOD (309 CFU/mL in tap water and 457 CFU/mL in minced beef) was obtained by Hassan et al. [89] who immobilized the capture antibody on magnetic beads and carried out a sandwich immunoassay using a AuNP-modified detection antibody (Figure 5A). The quantification of the bacteria was performed through the Hydrogen Evolution Reaction (HER) catalysed by the AuNP using chronoamperometry (applying +1.35 V for 60 s and then, −1.00 V for 100 s) and an SPCE as transducer. Wenchao Dou et al. used a nanocomposite consisting of gold-platinum core/shell nanoparticles, neutral red, and reduced graphene oxide (rGO-NR-Au@Pt) to develop different sensors for *E. coli* determination [90,91,92]. Using this nanocomposite to label the detection antibody, a sandwich-type immunosensor was developed by immobilizing the capture antibody on an AuNP/polyaniline-SPCE [90]. *E. coli* was quantified by taking advantage of the catalytic effect of the Au@Pt particles on the reduction of H_2_O_2_, achieving a high LOD of 2840 CFU/mL. Using a similar immunoassay and detection system (measurement of the reduction of H_2_O_2_ by CV on SPCE), Wenchao Dou et al. achieved a much better LOD (450 CFU/mL) by immobilizing the capture antibody on magnetic beads and using thionine as electron mediator [91]. They achieved an even lower LOD (91 CFU/mL), introducing HRP as a label (to catalyse the H_2_O_2_ reaction) in the rGO-NR-Au@Pt-detection antibody composite [92] (Figure 5B).

Among the label-free sensors for *E. coli* detection, it is worthy to note the one recently developed by Cimafonte et al. for drinking water [95]. It consisted of a sandwich-type immunosensor in which the capture antibody was immobilized with a suitable orientation on a SPAuE by a photochemical technique. The determination was performed by EIS using the [Fe(CN)_6_]^3−/4−^ redox probe, achieving a very low LOD of 30 CFU/mL.

Studies using screen-printed interdigitated electrodes were also found. An interesting one is the sensor developed by Xu et al. [97] that used a Prussian blue (PB)-modified screen-printed interdigitated gold microelectrode achieving a LOD of 190 CFU/g. An anti-E.coli antibody was immobilized on magnetic beads that were coated with polydopamine and modified with glucose oxidase (GOX) and AuNP. After the immunoreaction with the bacteria, a filtration was performed (through a paper with 0.8 µm pores) to separate the immunocomplex formed with the bacteria from the free nanocomposite-Ab. The analytical signal was recorded by amperometry dropping the filtered solution onto the PB-modified electrode together with a glucose solution (enzymatic substrate for GOX).

### 4.2. SPE-Based (Bio)Sensors for Biogenic Amines Detection

**Figure 6:**
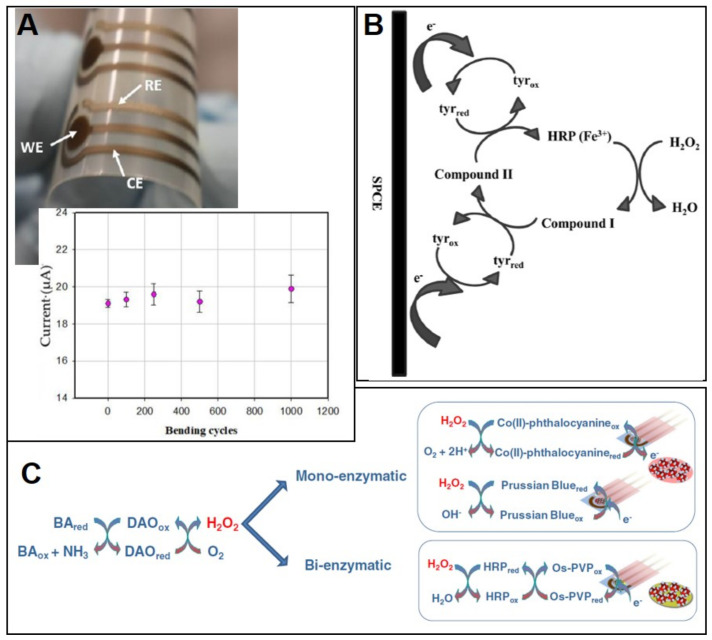
(**A**) Photograph of a flexible three-electrode SPE with silver working electrode used as transducer of an immunosensor for histamine, and flexibility test of that sensor (current intensity obtained after bending it). Reproduced from [98]). (**B**) Schematic representation of the enzymatic reaction occurring at the surface of the HRP/SPCE sensor for tyramine detection; Compound I and compound II are reaction intermediates (compound I (oxidation state +5) comprising a ferryl species (Fe^4+^=O) and a porphyrin radical cation; compound II (oxidation state +4) is formed by the first reduction of the porphyrin radical cation). Reproduced from [113] with permission from Wiley. (**C**) Scheme of the enzymatic and electrochemical reaction occurring on DAO-MB mono- and bi-enzymatic sensors for biogenic amines (BAs) detection. Reproduced from [117] from Springer 2016.

As mentioned before, histamine is the main BA. This explains the large number of sensors developed for its determination when compared to those developed for the other BAs. The most frequently reported SPE-based sensors for the determination of BAs in food samples (mainly fish) are enzymatic, although some immunosensors [98,99] or sensors based on the electroactivity of the amines [100,101] can also be found (Table 4). An interesting example among the immunosensors for histamine determination is the one recently developed by Shkodra et al. [98] using a flexible SPE with a WE made of a silver polymeric paste. The three-electrode cell is screen-printed on a polyethylene terephthalate (PET) flexible substrate to obtain a sensor that withstands frequent bending without signal loss (Figure 6A). To perform the immunoassay, an anti-histamine antibody is immobilized on the WE, previously modified with oxygen plasma-treated carbon nanotubes. Then, the competitive immunoassay is carried out using HRP-labelled histamine to compete with the histamine of the sample. Using 3,3′,5,5′-tetramethylbenzidine (TMB) as enzymatic substrate and chronoamperometry as detection technique, a sensor with a very low LOD (0.022 nM) and a high selectivity (tested using other BAs (cadaverine, putrescine, and tyramine) was obtained.

Among the large number of enzymatic sensors reported for the determination of BAs in food, most are based on the use of the enzymes monoamine oxidase (MAO) or diamine oxidase (DAO). These enzymes catalyse the oxidation of BAs, producing hydrogen peroxide [102,105,106,116,117,118]. The detection in these sensors is usually carried out by amperometric techniques and the use of redox mediators, such as [Fe(CN)_6_]^3−/4−^ [103], ferrocene [104,112], or tetrathiafulvalene (TTF) [106,107,115,116] is very common. The use of these mediators decreases the detection potential, improving the selectivity of the sensor. An interesting work was reported by S. Leonardo et al. in which different mono- (DAO) and bienzymatic (DAO and HRP) sensors using magnetic beads and different mediators (Co(II)-phthalocyanine (CoPh), Prussian Blue (PB), and Os-polyvinylpyridine (Os-PVP)) were developed and compared [117] (Figure 6C). Although calibration curves for histamine, putrescine and cadaverine were obtained for each one of the sensors (DAO-MB/CoPh-SPCE, DAO-MB/PB-SPCE, and DAO-MB/Os-PVP-HRP/SPCE), obtaining LODs from 0.47 µM to 5.13 µM, the one that included Prussian Blue as mediator was chosen for the determination of BAs in sea bass.

The use of nanomaterials is also frequent: single or multi-walled carbon nanotubes [104,109], graphene [108], nanoparticles [110,111,114,116], or the combination of different kind of nanomaterials [105]. An example is the sensor developed by Pérez et al. [104] that combines the use of two enzymes, DAO and horseradish peroxidase (HRP), with MWCNT and ferrocene as mediator. In this case, an SPCE with two WEs was used: one contained the enzymes immobilized on a polysulfone/MWCNT/ferrocene membrane and the other only contained the membrane. With this strategy, the response towards any electroactive species present in the samples that could interfere in the determination is eliminated, improving the selectivity of the sensor.

Sensors based on DAO and MAO or oxidase enzymes such as putrescine oxidase or plasma amine oxidase sometimes show problems regarding selectivity when just one biogenic amine is the target analyte [102,106,107,112,121]. Since for many food applications the objective is obtain the BAI (biogenic amine index), this fact could be not a problem and indeed, several sensors based on DAO and MAO are focused on the quantification of the total amount of BAs [117,118,119,120]. With the aim of obtaining more selective enzymatic sensors, the use of other enzymes as recognition element has been reported: e.g., tyrosinase [109] and histamine dehydrogenase [115]. A noteworthy example of a selective SPE-based enzymatic sensor for tyramine is the one developed by Calvo-Pérez et al. [113]. In this work HRP was used as recognition element for tyramine, which is not among the common substrates for this enzyme. The recognition of tyramine through HRP is based on the oxidation of the –OH group present in the molecular structure of tyramine (Figure 6B). Two immobilization procedures were assessed: (i) cross-linking with glutaraldehyde and bovine serum albumin and (ii) mixing the carbon ink used for screen-printing the WE with HRP. Since the second procedure was easier and provided better reproducibility, the sensor obtained in that way was the chosen for its application in real samples. A high selectivity of this HRP-sensor was demonstrated; no response was observed when calibration curves for other BAs (putrescine, cadaverine, histamine, tryptamine, spermine and spermidine were evaluated) in the same concentration range than for tyramine were performed. Another approach to greatly improve the selectivity is to add a separation step before the measurement with the sensor as reported by Li et al. [110]. In this work, a sensor based on an SPCE modified with a conductive polymer (PEDOT.PSS), AuNP and 1-methyl-4-mercaptopyridine (1m-4-MP) was developed to detect tyramine using DPV as technique detection. Before the electrochemical sensing, a sample was treated through a solid-phase extraction based on MIP technology (using a MIP synthetized with methacrylic acid as monomer). The combination of the MIP-based solid phase extraction with the 1-m-4-MP/AuNP/PEDOT:PSS/SPCE provided a LOD of 2.31 nM.

## 5. Conclusions

Nowadays, food safety is a key concern because it is directly related to public health. Therefore, the development of methods that allow rapid and on-site analysis has gained special relevance in food safety and quality assurance. Disposable electrodes, such as screen-printed electrodes (SPEs), have attracted attention worldwide since they allow the development of easy-to-handle and cost-efficient biosensors. The easy mass-production of reproducible SPEs allows the use of SPE-based sensors as one-shot devices. Besides the concern from the food industry and public-health-related administration about food safety and quality, the growing consumer concern about the security and healthiness of the food they eat enormously increases the interest in point-of-need sensors that can be used by untrained people.

Although there are many published papers on biosensors for food applications, the number of those commercially available is scare since the knowledge transfer from research laboratories to the market is hard. The main challenge for the commercialization of biosensors (for any kind of application) is often the low stability of their recognition element since they are biological compounds that requires special storage conditions. In the case of biosensors for food applications, another important difficulty is related to the sample since it is usually solid, and the measurements normally have to be performed in aqueous medium. This is an important limitation when compared with biosensors for clinical application that are typically applied to bodily fluids. Although the development of multiplex biosensors is increasing, multi-analyte detection is still a big challenge. In the case of food sensors, this is a key issue since, for example, a biosensor can be able to selectively determine a single bacteria serotype (i.e., *S. typhimurium*) but does not provide any information about the presence of other serotypes that can also be harmful. In those cases, the selection of a biological recognition able to detect several serotypes (or kinds of analytes) or the design of a multiplex devices is of paramount importance.

Therefore, it is obvious that biosensors cannot replace the conventional methods (e.g., PCR or HPLC-MS), since these show better features in terms of accuracy, selectively, sensitivity, or multi-analyte detection ability. However, the advantages of SPE-based biosensors mentioned in this review make them exceptional devices for on-site screening. The hard work of electroanalytical researchers to make portable sensors a suitable alternative to the centralized analysis, together the great advances in digital communication networks is leading to promising tools for food control and analysis. Nowadays, in a growing number of situations, it is much more advantageous to have simple tools for fast and on-site screening than sophisticated instrumentation in centralized laboratories.

## Figures and Tables

**Table 1:** Classification and basic information of the eight most common biogenic amines. Adapted from [52] with permission from Elsevier.

Classification	Name	Molecular Formula	Structure	Molecular Weight (g/mol)
Heterocyclic	Histamine (HIS)	C_5_H_9_N_3_	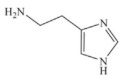	111.15
	Tryptamine (TRYP)	C_10_H_12_N_2_	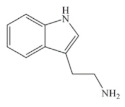	160.21
Aromatic	Phenylethylamine (PHEN)	C_8_H_11_N	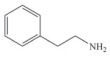	121.18
	Tyramine (TYR)	C_8_H_11_NO	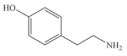	137.18
Aliphatic	Spermidine (SPD)	C_10_H_26_N_4_	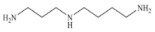	145.25
	Spermine (SPM)	C_7_H_19_N_3_	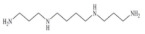	202.34
	Cadaverine (CAD)	C_5_H_14_N_2_	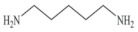	102.18
	Putrescine (PUT)	C_4_H_12_N_2_	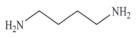	88.15

**Table 2:** SPE-based sensors for *Salmonella* and *Listeria* detection in foods.

Serotype	Sensor Construction	Detect. Tech.	Conc. Range	LOD	Analysis Time	Sample	Ref.
*Salmonella* Pullorum and *Salmonella* Gallinarum	Immunoassay; HRP as indirect label; ERGO/PVA-PDMS/SPCE	CV	10–10^9^ CFU/mL	1.61 CFU/mL	≈31 min	Chicken, eggs	[70]
*Salmonella* Pullorum and *Salmonella* Gallinarum	Immunoassay (sandwich); HRP as label; IL/Ab/AuNP/SPCE	CV	10^4^–10^9^ CFU/mL	3 × 10^3^ CFU/mL	≈81 min	Chicken, eggs	[71]
*Salmonella* Typhimurium	Immunoassay (sandwich); Ab-coated MB; Ag measurement; Avidin-SPCE	DPASV	10–10^6^ CFU/mL	12.6 CFU/mL	≈105 min	Milk, green bean sprouts, eggs	[81]
*Salmonella* Typhimurium	Immunoassay; Au-coated MB/SAM/Ab; CdSNP as label; SPCE	SWASV	10–10^6^ cell/mL	13 cells/mL	≈40 min	Milk	[68]
*Salmonella* Typhimurium	Immunoassay (sandwich); Ab on MB-MWCNT-Methylene Blue (which is the label); Avidin-SPCE	DPV	10–10^6^ CFU/mL in buffer and milk	7.9 CFU/mL in buffer; 17.3 CFU/mL in milk	≈55 min	Milk	[74]
*Salmonella* Typhimurium	Immunoassay (sandwich); Capture Ab on MB; AuNP as label; SPCE	DPV	10^3^–10^6^ cell/mL	143 cells/mL	≈95 min	Milk	[69]
*Salmonella* Typhimurium	Immunoassay; Label free; Ferrocyanide measurement; rG-GO/SPCE	EIS	−	10^1^ CFU/mL in samples	≈15 min	Water, orange juice	[72]
*Salmonella* Typhimurium	Aptasensor; Label free; diazonium salt- modified SPCE	EIS	10–10^8^ CFU/mL	6 CFU/mL	≈45 min	Apple juice	[73]
*Salmonella* Typhimurium	Paper-based immunoassay (sandwich); AuNP as label; SPCE	C	10–10^8^ CFU/mL	10 CFU/mL	≈35 min	Water	[76]
*Salmonella* Typhimurium	Immunoassay (sandwich); HRP as label; SPAuE	CA	10–10^7^ CFU/mL	≈20 CFU/mL	≈150 min	Chicken	[82]
*Salmonella* Typhimurium	Immunoassay (sandwich); HRP as label; SAM/Protein A/SPAuE	CA	−	10 CFU/mL	≈125 min	Milk	[62]
*Salmonella* (no serotype)	Immunoassay (sandwich); Capture Ab on MB; QD (CdTe) dendron as label; BiSPCE	SWASV	−	4 CFU/mL	≈80 min	Milk	[77]
*Salmonella* Typhimurium	Immunoassay; Label free; SAM/GA/Ab/2-SPAuE	EIS	10^3^–10^8^ CFU/mL	10^3^ CFU/mL	≈20 min	Milk	[79]
*Salmonella.* Typhimurium (and *E. coli* O157:H7)	Immunoassay (sandwich); Capture biotinylated Ab on stretavidin-MB; GOX-as label; SP-IDME (gold)	EIS	10^2^–10^6^ CFU/mL for both	1.66 × 10^3^ CFU/mL (3.90 × 10^2^ CFU/mL for *E. coli*)	≈180 min	Chicken carcass (ground beef for *E. coli*)	[83]
*Salmonella* Typhimurium and *Salmonella aureus* (and *E. coli*)	Antimicrobial petide melittin on MB; SP-IDME (silver)	EIS	10–10^4^ CFU/mL; 10–10^6^ CFU/mL (1–10^6^ CFU/mL for *E. coli*)	10 CFU/mL for both (1 CFU/mL for *E. coli*)	≈30 min	Water, apple juice	[84]
*Salmonella* Pullorum and *Salmonella* Gallinarum	Immunoassay (sandwich); Capture Ab on AuNP-modified MB (SiO_2_/Fe_3_O_4_); HRP as label; 4-SPCE	CV	10^2^–10^6^ CFU/mL	32 CFU/mL	≈70 min	Chicken	[85]
*Salmonella* Typhimurium	Immunoassay (sandwich); Capture Ab on MB; AuNP as label; µFD-8-SPCE	DPV	10.0–100.0 cell/mL in milk	7.7 cells/mL	≈75 min	Milk	[75]
*Salmonella* (no serotype)	Immunoassay (sandwich); HRP as label; 96-well SPCE plate	IPA	5 × 10^6^–5 × 10^8^ CFU/mL	2 × 10^6^ CFU/mL	≈100 min	Pork, chicken, beef	[80]
*Salmonella* (no serotype) (Multiplexed: *E. coli*, *Campylobacter*)	Immunoassay (sandwich); specific nanolabel for each specie (CuS, CdS, PbS); MWCNT-PAH/SPCE	SWASV	10^3^–5 × 10^5^ cell/mL	400 cells/mL for *Salmonella* and *Campylobacter*; 800 cells/mL for *E. coli*	≈70 min	Milk	[78]
*Listeria monocytogenes*	Immunoassay (sandwich); Ab capture on MB; Ab detection/urease (as label) modified AuNP; SP-IDE (gold)	EIS	1.9 × 10^3^–1.9 × 10^6^ CFU/mL	1.6 × 10^3^ CFU/mL	≈115 min	Lettuce	[86]
*Listeria innocua* Serovar 6b	Label-free; Bacteriophage endolysin CBD500 covalent immobilized on SPAuE	EIS	10^4^–10^9^ CFU/mL	1.1 × 10^4^ CFU/mL	≈25 min	Milk	[87]

4-SPCE: screen-printed carbon electrode with 4 working electrodes; 2-SPE: screen-printed electrode with 2 working electrodes; 8-SPCE: screen-printed carbon electrode with 8 working electrodes; µFD: microfluidic device; Ab: antibody; AP: alkaline phosphatase; AuNP: gold nanoparticles; BiSPCE: Bi film-modified screen-printed carbon electrode; C: conductometry; CA: chronoamperometry; CdSNP: CdS nanoparticles; CV: cyclic voltammetry; DPASV: differential pulse anodic stripping voltammetry; DPV: differential pulse voltammetry; EIS: electrochemical impedance spectroscopy; ERGO: electrochemically reduced graphene oxide; GA: Glutaraldehyde; HRP: horseradish peroxidase; GOX: glucose oxidase; IL: ionic liquid; IPA: intermittent pulse amperometry; MB: magnetic beads; MWCNT: multiwalled carbon nanotube; n.r: not reported; LSV: linear sweep voltammetry; PAH: polyallylamine; PDMS: polydimethylsiloxane; PVA: polyvinyl alcohol; QD: Quantum Dot; rG-GO: reduced graphene-graphene oxide; SPAuE: screen-printed gold electrode; SPCE: screen-printed carbon electrode; SP-IDME: screen-printed interdigitated electrode; SWASV: square wave anodic stripping voltammetry.

**Table 3:** SPE-based sensors for *E. coli* O157:H7 detection in foods.

Sensor Construction	Detect. Tech.	Conc. Range	LOD	Analysis Time	Sample	Ref.
Immunoassay (sandwich); HRP as label; AuNP/FeDC-SPCE	CA	10^2^ to 10^7^ CFU/mL	600 CFU/mL	≈35 min	Milk	[88]
Immunoassay (sandwich); Ab capture on MB; AuNP as label (catalysing HER); SPCE	CA	10^2^–10^5^ CFU/mL in samples	309 CFU/mL in tap water, 457 CFU/mL in minced beef	≈70 min	Water, minced beef	[89]
Immunoassay (sandwich); rGO-NR-Au@Pt nanocomposite-detection Ab (measurement of H_2_O_2_ reduction); AuNP/PANI-SPCE	CV	8.9 × 10^3^–8.9 × 10^9^ CFU/mL	2840 CFU/mL	≈110 min	Milk, pork	[90]
Immunoassay (sandwich); Capture Ab on MB; rGO-NR-Au@Pt nanocomposite-detection Ab (measurement of H_2_O_2_ reduction); Thionine as mediator; SPCE	CV	4 × 10^3^–4 × 10^8^ CFU/mL	450 CFU/mL	≈115 min	Milk, pork	[91]
Immunoassay (sandwich); Capture Ab on MB; rGO-NR-Au@Pt nanocomposite HRP-modified detection-Ab; HRP as label; Thionine as mediator; 4-SPCE	CV	4 × 10^2^–4 × 10^8^ CFU/mL	91 CFU/mL	≈135 min	Milk, pork	[92]
Immunoassay; Label-free (measurement of Fe(CN)_6_^3−/4−^); AuNP-SPCE	CV	1.19 × 10^3^–1.19 × 10^9^ CFU/mL	594 CFU/mL	≈55 min	Milk powder	[93]
Immunoassay; Label-free (measurement of Fe(CN)_6_^3−/4−^); AuNP/PANI-SPCE	DPV	4 × 10^4^–4 × 10^9^ CFU/mL	7980 CFU/mL	≈45 min	Milk	[94]
Immunoassay (sandwich); Ab photochemical immobilization; Label free; SPAuE	EIS	10^2^–10^3^ CFU/mL in drinking water	30 CFU/mL	≈70 min	Drinking water	[95]
Immunoassay; Capture Ab on MB; Label free; SP-IDME of gold	EIS	10^4^–10^7^ CFU/mL	10^4.45^ CFU/mL	≈60 min	Ground beef	[96]
Immunoassay; Ab on AuNP/MB-GOX@PDA; Filtration step; GOX as label; Prussian Blue-modified SP-IDME of gold	A	10^3^–10^6^ CFU/g in ground beef	190 CFU/g	≈75 min	Ground beef	[97]

4-SPCE: screen-printed carbon electrode with 4 working electrodes; A: amperometry; Ab: antibody; AuNP: gold nanoparticles; CA: chronoamperometry; CV: cyclic voltammetry; DPV: differential pulse voltammetry; EIS: electrochemical impedance spectroscopy; FeDC: ferrocene dicarboxylic acid; HER: hydrogen evolution reaction; HRP: horseradish peroxidase; ITO: indium tin oxide; MB: magnetic beads; NP: nanoparticles; NR: neutral red; PANI: polyaniline; PDA: polydopamine; rGO: reduced graphene oxide; SPAuE: screen-printed gold electrode; SPE: screen-printed electrode; SP-IDME: screen-printed interdigitated microelectrode.

**Table 4:** SPE-based sensors for biogenic amines detection in food samples.

Biogenic Amines	Sensor Construction	Detect. Tech.	Conc. Range	LOD	Analysis Time	Sample	Ref.
HIS	Rhenium (IV) oxide-SPCE	A	4.5–90 µM	1.8 µM	≈3 min	Fish sauce	[100]
HIS	Nafion/Cu_3_(PO_4_)_2_NP/SPCE	A	0.045–4.5 mM	0.027 mM	≈3 min	Fish	[101]
HIS	Immunoassay (competitive); Histamine labelled with HRP; Capture Ab on SWCNT/SPE (flexible with a silver WE)	CA	0.045–450 nM	0.022 nM	≈140 min	Fish	[98]
HIS	Immunoassay (competitive); HRP-labelled detection Ab; Histamine-ovalbumin conjugate on PB/chitosan/AuNP/SPCE	CV	0.09–900 µM	0.01 nM	≈130 min	Fish	[99]
HIS	DAO on SPCE	CA	9–675 µM	4.5 µM	≈1 min	Fish (hake, mackerel)	[102]
HIS	DAO on SPCE; [Fe(CN)_6_]^3−^ in solution as mediator	CA	45–675 µM	8.7 µM	≈7 min	Fish (tuna, mackerel)	[103]
HIS	DAO and HRP on polysulfone/ MWCNT/ferrocene membrane/SPCE; SPCE with two WE, ferrocene as mediator	A	0.3–20 µM	0.17 µM	≈2 min	Fish (anchovy, tuna, sardine, mackerel, shrimp, grater weever)	[104]
HIS	DAO on PtNP/rGO/chitosan/SPCE	A	0.1–300 µM	25.4 nM	≈2 min	Fish (carp, tench, catfish, perch)	[105]
PUT	MAO on TTF-SPCE; TTF as mediator	A	16–101 µM	17.2 µM	≈2 min	Anchovy, Courgette	[106]
PUT	PUO on TTF-SPCE; TTF as mediator	A	10–74 µM	10.1 µM	≈2 min	Octopus, courgette	[107]
TYR	DAO on GO/PVF-modified SPCE	A	0.99–120 µM	0.41 µM	≈2 min	Cheese	[108]
	MAO on GO/PVF-modified SPCE		0.9–110 µM	0.61 µM			
TYR	Ty on SWCNT/SPCE	A	5–180 µM	0.62 µM	≈2 min	Fish	[109]
TYR	1-methyl-4-mercaptopyridine/AuNP/PEDOT:PSS/SPCE	DPV	5–100 nM	2.31 nM	≈6 min	Milk	[110]
TYR	Nafion/Ty/Fe_3_O_4_-chitosan/poly-L-lysine/SPCE	A	0.49–63 µM	0.075 µM	≈2 min	Cheese	[111]
TYR	PAO on SPCE (hydroxymethylferrocene in cell solution as mediator)	A	2–164 µM	2.0 µM	≈2 min	Cheese	[112]
TYR	HRP on SPCE	A	2–456 µM	2.1 µM	≈2 min	Cheese	[113]
HIS	DAO on PB/ITO nanoparticles/SPCE	A	6.0–690 µM	1.9 µM	≈2 min	Cheese	[114]
CAD	MAO on PB/ITO nanoparticles/SPCE		3–1000 µM	0.9 µM			
HIS	HMD and PUO respectively on TTF-SPCE (with 4 WE); TTF as mediator	A	−	8.1 µM	≈2 min	Octopus	[115]
PUT			−	10 µM			
PUT	MAO (for PUT) or MAO/AuNPs (for PUT and CAD) on TTF-SPCE (with two WE); TTF as mediator	A	9.9–74.1 µM	9.9 µM	≈2 min	Octopus	[116]
CAD			19.6–107.1 µM	19.9 µM			
Total biogenic amines (calibration with HIS, PUT, CAD)	DAO on MB; PB-SPCE	CA	0.01–1 mM for HIS, PUT, CAD	4.8 µM for HIS; 0.9 µM for PUT; 0.67 µM for CAD	≈15 min	Fish (sea bass)	[117]
Total biogenic amines (calibration with HIS)	DAO and HRP on aryl diazonium salt/SPCE	A	0.2–1.6 µM	0.18 µM	≈2 min	Fish (anchovy)	[118]
Total biogenic amines (calibration with PUT)	DAO on polyazetidine prepolimer/SPE (with two WE of gold)	A	8–227 µM	2.3 µM	≈2 min	Wine, beer	[119]
Total biogenic amines (calibration with CAD, PUT, TYR, HIS)	Nafion/DAO/MnO_2_-SPCE (MnO_2_ as mediator)	A	1–50 µM for CAD and PUT; 10–300 µM for TYR and HIS	0.3 µM for CAD and PUT; 3.0 µM for TYR and HIS	≈5 min	Chicken meat	[120]

A: amperometry; AuNP: gold nanoparticles; BSA: bovine serum albumin; CA: chronoamperometry; CAD: cadaverine; CV: cyclic voltammetry; DAO: diamine oxidase; DPV: differential pulse voltammetry; GO: graphene oxide; HIS: histamine; HMD: histamine dehydrogenase; HRP: horseradish peroxidase; ITO: indium tin oxide; MAO: monoamine oxidase; MB: magnetic beads; MWCNT: multi-walled carbon nanotubes; NP: nanoparticles; PAO: plasma amine oxidase; PB: Prussian blue; PEDOT:PSS: poly(3,4-ethylenedioxythiophene):poly-styrene sulfonate: PtNP: platinum nanoparticles; PUO: putrescine oxidase; PUT: putrescine; PVF: polyvinylferrocene; rGO: reduced graphene oxide; SPCE: screen-printed carbon electrode; SPE: screen-printed electrode; SWCNT: single-walled carbon nanotubes; TTF: tetrathiafulvalene; Ty: tyrosinase; TYR: tyramine; WE: working electrode.

## References

[1] World Health Organization https://www.who.int/news-room/fact-sheets/detail/food-safety.

[2] Den Besten H.M.W., Wells-Bennik M.H.J., Zwietering M.H. (2018). Natural Diversity in Heat Resistance of Bacteria and Bacterial Spores: Impact on Food Safety and Quality. Annu. Rev. Food Sci. Technol..

[3] Abraham A., Al-Khaldi S., Assimon S.A., Beuadry C., Benner R.A., Bennett R., Binet R., Cahill S.M., Burkhardt W. (2012). Bad Bud Book. Handbook of Foodborne Pathogenic Microorganisms and Natural Toxins Introduction.

[4] Naila A., Flint S., Fletcher G., Bremer P., Meerdink G. (2010). Control of biogenic amines in food—Existing and emerging approaches. J. Food Sci..

[5] Velusamy V., Arshak K., Korostynska O., Oliwa K., Adley C. (2010). An overview of foodborne pathogen detection: In the perspective of biosensors. Biotechnol. Adv..

[6] Zhao X., Lin C.W., Wang J., Oh D.H. (2014). Advances in rapid detection methods for foodborne pathogens. J. Microbiol. Biotechnol..

[7] European Food Safety Authority (EFSA) (2011). BIOHAZ Scientific Opinion on risk based control of biogenic amine formation in fermented foods. EFSA J..

[8] Biji K.B., Ravishankar C.N., Venkateswarlu R., Mohan C.O., Gopal T.K.S. (2016). Biogenic amines in seafood: A review. J. Food Sci. Technol..

[9] Jairath G., Singh P.K., Dabur R.S., Rani M., Chaudhari M. (2015). Biogenic amines in meat and meat products and its public health significance: A review. J. Food Sci. Technol..

[10] Papageorgiou M., Lambropoulou D., Morrison C., Kłodzińska E., Namieśnik J., Płotka-Wasylka J. (2018). Literature update of analytical methods for biogenic amines determination in food and beverages. TrAC Trends Anal. Chem..

[11] Köse S., Kaklikkaya N., Koral S., Tufan B., Buruk K.C., Aydin F. (2011). Commercial test kits and the determination of histamine in traditional (ethnic) fish products-evaluation against an EU accepted HPLC method. Food Chem..

[12] Mishra R.K., Nunes G.S., Souto L., Marty J.L. (2018). Screen printed technology—An application towards biosensor development. Encyclopedia of Interfacial Chemistry.

[13] Smart A., Crew A., Pemberton R., Hughes G., Doran O., Hart J.P. (2020). Screen-printed carbon based biosensors and their applications in agri-food safety. TrAC Trends Anal. Chem..

[14] Vasilescu A., Nunes G., Hayat A., Latif U., Marty J.L. (2016). Electrochemical affinity biosensors based on disposable screen-printed electrodes for detection of food allergens. Sensors.

[15] Díaz-Cruz J.M., Serrano N., Pérez-Ràfols C., Ariño C., Esteban M. (2020). Electroanalysis from the past to the twenty-first century: Challenges and perspectives. J. Solid State Electrochem..

[16] Roberts G., Age S., Simon S. (2006). History’s Influence on Screen Printing’s Future Explore How Screenprinting’s Past Will Shape Its Future. *Screen Print*. https://www.screenweb.com/content/historys-influence-screen-printings-future.

[17] Couto R.A.S., Lima J.L.F.C., Quinaz M.B. (2016). Recent developments, characteristics and potential applications of screen-printed electrodes in pharmaceutical and biological analysis. Talanta.

[18] Arduini F., Micheli L., Moscone D., Palleschi G., Piermarini S., Ricci F., Volpe G. (2016). Electrochemical biosensors based on nanomodified screen-printed electrodes: Recent applications in clinical analysis. Trends Anal. Chem..

[19] Rama E.C., Costa-García A. (2016). Screen-printed Electrochemical Immunosensors for the Detection of Cancer and Cardiovascular Biomarkers. Electroanalysis.

[20] Li M., Li Y.T., Li D.W., Long Y.T. (2012). Recent developments and applications of screen-printed electrodes in environmental assays-A review. Anal. Chim. Acta.

[21] Hayat A., Marty J.L. (2014). Disposable screen printed electrochemical sensors: Tools for environmental monitoring. Sensors.

[22] Cano-Raya C., Denchev Z.Z., Cruz S.F., Viana J.C. (2019). Chemistry of solid metal-based inks and pastes for printed electronics–A review. Appl. Mater. Today.

[23] Yáñez-Sedeño P., Campuzano S., Pingarrón J.M. (2019). Electrochemical (bio)sensors: Promising tools for green analytical chemistry. Curr. Opin. Green Sustain. Chem..

[24] Gałuszka A., Migaszewski Z., Namieśnik J. (2013). The 12 principles of green analytical chemistry and the SIGNIFICANCE mnemonic of green analytical practices. TrAC Trends Anal. Chem..

[25] Sanati A., Jalali M., Raeissi K., Karimzadeh F., Kharaziha M., Mahshid S.S., Mahshid S. (2019). A review on recent advancements in electrochemical biosensing using carbonaceous nanomaterials. Microchim. Acta.

[26] Metrohm DropSens http://www.dropsens.com/.

[27] Micrux Technologies http://www.micruxfluidic.com/.

[28] Pine Research https://pineresearch.com/.

[29] Gwent Group http://www.gwent.org/.

[30] PalmSens https://www.palmsens.com/.

[31] Rusens http://www.rusens.com/indexeng.html.

[32] Putzbach W., Ronkainen N.J. (2013). Immobilization techniques in the fabrication of nanomaterial-based electrochemical biosensors: A review. Sensors (Basel).

[33] Antuña-Jiménez D., González-García M.B., Hernández-Santos D., Fanjul-Bolado P. (2020). Screen-printed electrodes modified with metal nanoparticles for small molecule sensing. Biosensors.

[34] Duffy G.F., Moore E.J. (2017). Electrochemical Immunosensors for Food Analysis: A Review of Recent Developments. Anal. Lett..

[35] Windmiller J.R., Bandodkar A.J., Parkhomovsky S., Wang J. (2012). Stamp transfer electrodes for electrochemical sensing on non-planar and oversized surfaces. Analyst.

[36] Mishra R.K., Hubble L.J., Martín A., Kumar R., Barfidokht A., Kim J., Musameh M.M., Kyratzis I.L., Wang J. (2017). Wearable flexible and stretchable glove biosensor for on-site detection of organophosphorus chemical threats. ACS Sens..

[37] Desmet C., Marquette C.A., Blum L.J., Doumèche B. (2016). Paper electrodes for bioelectrochemistry: Biosensors and biofuel cells. Biosens. Bioelectron..

[38] Moro G., Bottari F., Van Loon J., Du Bois E., De Wael K., Moretto L.M. (2019). Disposable electrodes from waste materials and renewable sources for (bio)electroanalytical applications. Biosens. Bioelectron..

[39] Neves M.M.P.S., González-García M.B., Hernández-Santos D., Fanjul-Bolado P. (2014). Screen-Printed Electrochemical 96-Well Plate: A High-Throughput Platform for Multiple Analytical Applications. Electroanalysis.

[40] Piermarini S., Micheli L., Ammida N.H.S., Palleschi G., Moscone D. (2007). Electrochemical immunosensor array using a 96-well screen-printed microplate for aflatoxin B1 detection. Biosens. Bioelectron..

[41] Thévenot D.R., Toth K., Durst R.A., Wilson G.S. (2001). Electrochemical biosensors: Recommended definitions and classification1International Union of Pure and Applied Chemistry: Physical Chemistry Division, Commission I.7 (Biophysical Chemistry); Analytical Chemistry Division, Commission V.5 (Electroanalytical). Biosens. Bioelectron..

[42] Sharma H., Mutharasan R. (2013). Review of biosensors for foodborne pathogens and toxins. Sens. Actuators B Chem..

[43] Ronkainen N.J., Halsall H.B., Heineman W.R. (2010). Electrochemical biosensors. Chem. Soc. Rev..

[44] Sassolas A., Blum L.J., Leca-Bouvier B.D. (2012). Immobilization strategies to develop enzymatic biosensors. Biotechnol. Adv..

[45] Crapnell R.D., Hudson A., Foster C.W., Eersels K., van Grinsven B., Cleij T.J., Banks C.E., Peeters M. (2019). Recent advances in electrosynthesized molecularly imprinted polymer sensing platforms for bioanalyte detection. Sensors (Switzerland).

[46] Tudorache M., Bala C. (2007). Biosensors based on screen-printing technology, and their applications in environmental and food analysis. Anal. Bioanal. Chem..

[47] Ricci F., Adornetto G., Palleschi G. (2012). A review of experimental aspects of electrochemical immunosensors. Electrochim. Acta.

[48] Cesewski E., Johnson B.N. (2020). Electrochemical biosensors for pathogen detection. Biosens. Bioelectron..

[49] Wang Y., Duncan T.V. (2017). Nanoscale sensors for assuring the safety of food products. Curr. Opin. Biotechnol..

[50] Silva N.F.D., Neves M.M.P.S., Magalhães J.M.C.S., Freire C., Delerue-Matos C. (2020). Emerging electrochemical biosensing approaches for detection of *Listeria monocytogenes* in food samples: An overview. Trends Food Sci. Technol..

[51] Silva N.F.D., Magalhães J.M.C.S., Freire C., Delerue-Matos C. (2018). Electrochemical biosensors for *Salmonella*: State of the art and challenges in food safety assessment. Biosens. Bioelectron..

[52] Zhang Y.-J., Zhang Y., Zhou Y., Li G.-H., Yang W.-Z., Feng X.-S. (2019). A review of pretreatment and analytical methods of biogenic amines in food and biological samples since 2010. J. Chromatogr. A.

[53] Prabhakar P.K., Vatsa S., Srivastav P.P., Pathak S.S. (2020). A comprehensive review on freshness of fish and assessment: Analytical methods and recent innovations. Food Res. Int..

[54] European Food Safety Authority (EFSA) http://www.efsa.europa.eu/en/news/salmonella-most-common-cause-foodborne-outbreaks-european-union.

[55] European Food Safety Authority (EFSA) (2016). ECDC The European Union summary report on trends and sources of zoonoses, zoonotic agents and food-borne outbreaks in 2015. EFSA J..

[56] Bolton D.J. (2015). *Campylobacter* virulence and survival factors. Food Microbiol..

[57] Silva J., Leite D., Fernandes M., Mena C., Gibbs P.A., Teixeira P. (2011). *Campylobacter* spp. As a foodborne pathogen: A review. Front. Microbiol..

[58] Fabiani L., Delibato E., Volpe G., Piermarini S., De Medici D., Palleschi G. (2019). Development of a sandwich ELIME assay exploiting different antibody combinations as sensing strategy for an early detection of *Campylobacter*. Sens. Actuators B Chem..

[59] Allocati N., Masulli M., Alexeyev M.F., Di Ilio C. (2013). *Escherichia coli* in Europe: An overview. Int. J. Environ. Res. Public Health.

[60] Kaper J.B., Nataro J.P., Mobley H.L.T. (2004). Pathogenic *Escherichia coli*. Nat. Rev. Microbiol..

[61] Eng S.K., Pusparajah P., Ab Mutalib N.S., Ser H.L., Chan K.G., Lee L.H. (2015). *Salmonella*: A review on pathogenesis, epidemiology and antibiotic resistance. Front. Life Sci..

[62] Alexandre D.L., Melo A.M.A., Furtado R.F., Borges M.F., Figueiredo E.A.T., Biswas A., Cheng H.N., Alves C.R. (2018). A Rapid and Specific Biosensor for *Salmonella* Typhimurium Detection in Milk. Food Bioprocess Technol..

[63] Cinti S., Volpe G., Piermarini S., Delibato E., Palleschi G. (2017). Electrochemical biosensors for rapid detection of foodborne *Salmonella*: A critical overview. Sensors (Switzerland).

[64] Buchanan R.L., Gorris L.G.M., Hayman M.M., Jackson T.C., Whiting R.C. (2017). A review of *Listeria monocytogenes*: An update on outbreaks, virulence, dose-response, ecology, and risk assessments. Food Control.

[65] De Jong W.H.A., De Vries E.G.E., Kema I.P. (2011). Current status and future developments of LC-MS/MS in clinical chemistry for quantification of biogenic amines. Clin. Biochem..

[66] Ahmad W., Mohammed G.I., Al-Eryani D.A., Saigl Z.M., Alyoubi A.O., Alwael H., Bashammakh A.S., O’Sullivan C.K., El-Shahawi M.S. (2019). Biogenic Amines Formation Mechanism and Determination Strategies: Future Challenges and Limitations. Crit. Rev. Anal. Chem..

[67] Ordóñez J.L., Troncoso A.M., García-Parrilla M.D.C., Callejón R.M. (2016). Recent trends in the determination of biogenic amines in fermented beverages—A review. Anal. Chim. Acta.

[68] Freitas M., Viswanathan S., Nouws H.P.A., Oliveira M.B.P.P., Delerue-Matos C. (2014). Iron oxide/gold core/shell nanomagnetic probes and CdS biolabels for amplified electrochemical immunosensing of *Salmonella* typhimurium. Biosens. Bioelectron..

[69] Afonso A.S., Pérez-López B., Faria R.C., Mattoso L.H.C., Hernández-Herrero M., Roig-Sagués A.X., Maltez-da Costa M., Merkoçi A. (2013). Electrochemical detection of *Salmonella* using gold nanoparticles. Biosens. Bioelectron..

[70] Wang D., Dou W., Chen Y., Zhao G. (2014). Enzyme-functionalized electrochemical immunosensor based on electrochemically reduced graphene oxide and polyvinyl alcohol-polydimethylsiloxane for the detection of *Salmonella* pullorum & *Salmonella* gallinarum. RSC Adv..

[71] Fei J., Dou W., Zhao G. (2015). A sandwich electrochemical immunosensor for *Salmonella* pullorum and *Salmonella* gallinarum based on a screen-printed carbon electrode modified with an ionic liquid and electrodeposited gold nanoparticles. Microchim. Acta.

[72] Mutreja R., Jariyal M., Pathania P., Sharma A., Sahoo D.K., Suri C.R. (2016). Novel surface antigen based impedimetric immunosensor for detection of *Salmonella* typhimurium in water and juice samples. Biosens. Bioelectron..

[73] Bagheryan Z., Raoof J.B., Golabi M., Turner A.P.F., Beni V. (2016). Diazonium-based impedimetric aptasensor for the rapid label-free detection of *Salmonella* typhimurium in food sample. Biosens. Bioelectron..

[74] Ngoensawat U., Rijiravanich P., Surareungchai W., Somasundrum M. (2018). Electrochemical Immunoassay for *Salmonella* Typhimurium Based on an Immuno-magnetic Redox Label. Electroanalysis.

[75] De Oliveira T.R., Martucci D.H., Faria R.C. (2018). Simple disposable microfluidic device for *Salmonella* typhimurium detection by magneto-immunoassay. Sens. Actuators B Chem..

[76] Wonsawat W., Limvongjaroen S., Supromma S., Panphut W., Ruecha N., Ratnarathorn N., Dungchai W. (2020). A paper-based conductive immunosensor for the determination of *Salmonella* Typhimurium. Analyst.

[77] Murasova P., Kovarova A., Kasparova J., Brozkova I., Hamiot A., Pekarkova J., Dupuy B., Drbohlavova J., Bilkova Z., Korecka L. (2020). Direct culture-free electrochemical detection of *Salmonella* cells in milk based on quantum dots-modified nanostructured dendrons. J. Electroanal. Chem..

[78] Viswanathan S., Rani C., Ho J.A.H.A. (2012). Electrochemical immunosensor for multiplexed detection of food-borne pathogens using nanocrystal bioconjugates and MWCNT screen-printed electrode. Talanta.

[79] Farka Z., Juřík T., Pastucha M., Kovář D., Lacina K., Skládal P. (2016). Rapid Immunosensing of *Salmonella* Typhimurium Using Electrochemical Impedance Spectroscopy: The Effect of Sample Treatment. Electroanalysis.

[80] Delibato E., Volpe G., Stangalini D., De Medici D., Moscone D., Palleschi G. (2006). Development of SYBR-green real-time PCR and a multichannel electrochemical immunosensor for specific detection of *Salmonella* enterica. Anal. Lett..

[81] Pratiwi F.W., Rijiravanich P., Somasundrum M., Surareungchai W. (2013). Electrochemical immunoassay for *Salmonella* Typhimurium based on magnetically collected Ag-enhanced DNA biobarcode labels. Analyst.

[82] Salam F., Tothill I.E. (2009). Detection of *Salmonella* typhimurium using an electrochemical immunosensor. Biosens. Bioelectron..

[83] Xu M., Wang R., Li Y. (2016). Rapid detection of *Escherichia coli* O157:H7 and *Salmonella* Typhimurium in foods using an electrochemical immunosensor based on screen-printed interdigitated microelectrode and immunomagnetic separation. Talanta.

[84] Wilson D., Materón E.M., Ibáñez-Redín G., Faria R.C., Correa D.S., Oliveira O.N. (2019). Electrical detection of pathogenic bacteria in food samples using information visualization methods with a sensor based on magnetic nanoparticles functionalized with antimicrobial peptides. Talanta.

[85] Fei J., Dou W., Zhao G. (2015). A sandwich electrochemical immunoassay for Salmonella pullorum and *Salmonella* gallinarum based on a AuNPs/SiO_2_/Fe3O_4_ adsorbing antibody and 4 channel screen printed carbon electrode electrodeposited gold nanoparticles. RSC Adv..

[86] Wang D., Chen Q., Huo H., Bai S., Cai G., Lai W., Lin J. (2017). Efficient separation and quantitative detection of *Listeria monocytogenes* based on screen-printed interdigitated electrode, urease and magnetic nanoparticles. Food Control.

[87] Tolba M., Ahmed M.U., Tlili C., Eichenseher F., Loessner M.J., Zourob M. (2012). A bacteriophage endolysin-based electrochemical impedance biosensor for the rapid detection of *Listeria* cells. Analyst.

[88] Lin Y.H., Chen S.H., Chuang Y.C., Lu Y.C., Shen T.Y., Chang C.A., Lin C.S. (2008). Disposable amperometric immunosensing strips fabricated by Au nanoparticles-modified screen-printed carbon electrodes for the detection of foodborne pathogen *Escherichia coli* O157:H7. Biosens. Bioelectron..

[89] Hassan A.R.H.A.A., de la Escosura-Muñiz A., Merkoçi A. (2015). Highly sensitive and rapid determination of *Escherichia coli* O157:H7 in minced beef and water using electrocatalytic gold nanoparticle tags. Biosens. Bioelectron..

[90] Mo X., Wu Z., Huang J., Zhao G., Dou W. (2019). A sensitive and regenerative electrochemical immunosensor for quantitative detection of: *Escherichia coli* O157:H7 based on stable polyaniline coated screen-printed carbon electrode and rGO-NR-Au@Pt. Anal. Methods.

[91] Zhu F., Zhao G., Dou W. (2018). A non-enzymatic electrochemical immunoassay for quantitative detection of *Escherichia coli* O157:H7 using Au@Pt and graphene. Anal. Biochem..

[92] Zhu F., Zhao G., Dou W. (2018). Electrochemical sandwich immunoassay for *Escherichia coli* O157:H7 based on the use of magnetic nanoparticles and graphene functionalized with electrocatalytically active Au@Pt core/shell nanoparticles. Microchim. Acta.

[93] Huang Y., Wu Z., Zhao G., Dou W. (2019). A Label-Free Electrochemical Immunosensor Modified with AuNPs for Quantitative Detection of *Escherichia coli* O157:H7. J. Electron. Mater..

[94] Mo X., Zhao G., Dou W. (2018). Electropolymerization of Stable Leucoemeraldine Base Polyaniline Film and Application for Quantitative Detection of *Escherichia coli* O157:H7. J. Electron. Mater..

[95] Cimafonte M., Fulgione A., Gaglione R., Papaianni M., Capparelli R., Arciello A., Censi S.B., Borriello G., Velotta R., Ventura B. (2020). Della Screen printed based impedimetric immunosensor for rapid detection of *Escherichia coli* in drinking water. Sensors.

[96] Wang R., Lum J., Callaway Z., Lin J., Bottje W., Li Y. (2015). A label-free impedance immunosensor using screen-printed interdigitated electrodes and magnetic nanobeads for the detection of *E. coli* O157:H7. Biosensors.

[97] Xu M., Wang R., Li Y. (2016). An electrochemical biosensor for rapid detection of: *E. coli* O157:H7 with highly efficient bi-functional glucose oxidase-polydopamine nanocomposites and Prussian blue modified screen-printed interdigitated electrodes. Analyst.

[98] Shkodra B., Abera B.D., Cantarella G., Douaki A., Avancini E., Petti L., Lugli P. (2020). Flexible and printed electrochemical immunosensor coated with oxygen plasma treated SWCNTs for histamine detection. Biosensors.

[99] Dong X.X., Yang J.Y., Luo L., Zhang Y.F., Mao C., Sun Y.M., Lei H.T., Shen Y.D., Beier R.C., Xu Z.L. (2017). Portable amperometric immunosensor for histamine detection using Prussian blue-chitosan-gold nanoparticle nanocomposite films. Biosens. Bioelectron..

[100] Veseli A., Vasjari M., Arbneshi T., Hajrizi A., Švorc L., Samphao A., Kalcher K. (2016). Electrochemical determination of histamine in fish sauce using heterogeneous carbon electrodes modified with rhenium(IV) oxide. Sens. Actuators B Chem..

[101] Lee M.-Y., Wu C.-C., Sari M.I., Hsieh Y. (2018). A disposable non-enzymatic histamine sensor based on the nafion-coated copper phosphate electrodes for estimation of fish freshness. Electrochim. Acta.

[102] Torre R., Costa-Rama E., Lopes P., Nouws H.P.A., Delerue-Matos C. (2019). Amperometric enzyme sensor for the rapid determination of histamine. Anal. Methods.

[103] Torre R., Costa-rama E., Nouws H.P.A., Delerue-Matos C. (2020). Diamine oxidase-modified screen-printed electrode for the redox-mediated determination of histamine. J. Anal. Sci. Technol..

[104] Pérez S., Bartrolí J., Fàbregas E. (2013). Amperometric biosensor for the determination of histamine in fish samples. Food Chem..

[105] Apetrei I.M., Apetrei C. (2016). Amperometric biosensor based on diamine oxidase/platinum nanoparticles/graphene/chitosan modified screen-printed carbon electrode for histamine detection. Sensors.

[106] Henao-Escobar W., Domínguez-Renedo O., Alonso-Lomillo M.A., Arcos-Martínez M.J. (2013). A screen-printed disposable biosensor for selective determination of putrescine. Microchim. Acta.

[107] Henao-Escobar W., Domínguez-Renedo O., Alonso-Lomillo M.A., Cascalheira J.F., Dias-Cabral A.C., Arcos-Martínez M.J. (2015). Characterization of a Disposable Electrochemical Biosensor Based on Putrescine Oxidase from Micrococcus rubens for the Determination of Putrescine. Electroanalysis.

[108] Erden P.E., Erdoğan Z.Ö., Öztürk F., Koçoğlu İ.O., Kılıç E. (2019). Amperometric Biosensors for Tyramine Determination Based on Graphene Oxide and Polyvinylferrocene Modified Screen-printed Electrodes. Electroanalysis.

[109] Apetrei I.M., Apetrei C. (2015). The biocomposite screen-printed biosensor based on immobilization of tyrosinase onto the carboxyl functionalised carbon nanotube for assaying tyramine in fish products. J. Food Eng..

[110] Li Y., Hsieh C.H., Lai C.-W., Chang Y.-F., Chan H.-Y., Tsai C.-F., Ho J.A., Wu L. (2017). Tyramine detection using PEDOT:PSS/AuNPs/1-methyl-4-mercaptopyridine modified screen-printed carbon electrode with molecularly imprinted polymer solid phase extraction. Biosens. Bioelectron..

[111] Dalkıran B., Erden P.E., Kaçar C., Kılıç E. (2019). Disposable Amperometric Biosensor Based on Poly-L-lysine and Fe_3_O_4_ NPs-chitosan Composite for the Detection of Tyramine in Cheese. Electroanalysis.

[112] Calvo-Pérez A., Domínguez-Renedo O., Alonso-Lomillo M.A., Arcos-Martínez M.J. (2013). Disposable amperometric biosensor for the determination of tyramine using plasma amino oxidase. Microchim. Acta.

[113] Calvo-Pérez A., Domínguez-Renedo O., Alonso-Lomillo M.A., Arcos-Martínez M.J. (2013). Disposable Horseradish Peroxidase Biosensors for the Selective Determination of Tyramine. Electroanalysis.

[114] Kaçar C., Erden P.E., Dalkiran B., İnal E.K., Kiliç E. (2020). Amperometric biogenic amine biosensors based on Prussian blue, indium tin oxide nanoparticles and diamine oxidase—Or monoamine oxidase–modified electrodes. Anal. Bioanal. Chem..

[115] Henao-Escobar W., Román L.D.T.-D., Domínguez-Renedo O., Alonso-Lomillo M.A., Arcos-Martínez M.J. (2016). Dual enzymatic biosensor for simultaneous amperometric determination of histamine and putrescine. Food Chem..

[116] Henao-Escobar W., Domínguez-Renedo O., Asunción Alonso-Lomillo M., Julia Arcos-Martínez M. (2013). Simultaneous determination of cadaverine and putrescine using a disposable monoamine oxidase based biosensor. Talanta.

[117] Leonardo S., Campàs M. (2016). Electrochemical enzyme sensor arrays for the detection of the biogenic amines histamine, putrescine and cadaverine using magnetic beads as immobilisation supports. Microchim. Acta.

[118] Alonso-Lomillo M.A., Domínguez-Renedo O., Matos P., Arcos-Martínez M.J. (2010). Disposable biosensors for determination of biogenic amines. Anal. Chim. Acta.

[119] Di Fusco M., Federico R., Boffi A., MacOne A., Favero G., Mazzei F. (2011). Characterization and application of a diamine oxidase from Lathyrus sativus as component of an electrochemical biosensor for the determination of biogenic amines in wine and beer. Anal. Bioanal. Chem..

[120] Telsnig D., Kalcher K., Leitner A., Ortner A. (2013). Design of an Amperometric Biosensor for the Determination of Biogenic Amines Using Screen Printed Carbon Working Electrodes. Electroanalysis.

[121] Lange J., Wittmann C. (2002). Enzyme sensor array for the determination of biogenic amines in food samples. Anal. Bioanal. Chem..

